# Design, Fabrication, and Dynamic Analysis of a MEMS Ring Resonator Supported by Twin Circular Curve Beams

**DOI:** 10.3390/s24144499

**Published:** 2024-07-11

**Authors:** Ahmad Rahbar Ranji, Gnanesh Nagesh, Fangyan Sun, Mohammed Jalal Ahamed

**Affiliations:** Department of Mechanical, Automotive and Material Engineering, University of Windsor, Windsor, ON N9B 3P4, Canada

**Keywords:** MEMS, dynamic analysis, electrostatic force, FEM, pull-in voltage, ring resonator

## Abstract

In this paper, we present a compressive study on the design and development of a MEMS ring resonator and its dynamic behavior under electrostatic force when supported by twin circular curve beams. Finite element analysis (FEA)-based modeling techniques are used to simulate and refine the resonator geometry and transduction. In proper FEA or analytical modeling, the explicit description and accurate values of the effective mass and stiffness of the resonator structure are needed. Therefore, here we outlined an analytical model approach to calculate those values using the first principles of kinetic and potential energy analyses. The natural frequencies of the structure were then calculated using those parameters and compared with those that were simulated using the FEA tool ANSYS. Dynamic analysis was performed to calculate the pull-in voltage, shift of resonance frequency, and harmonic analyses of the ring to understand how the ring resonator is affected by the applied voltage. Additional analysis was performed for different orientations of silicon and assessing the frequency response and frequency shifts. The prototype was fabricated using the standard silicon-on-insulator (SOI)-based MEMS fabrication process and the experimental results for resonances showed good agreement with the developed model approach. The model approach presented in this paper can be used to provide valuable insights for the optimization of MEMS resonators for various operating conditions.

## 1. Introduction

Ring- and disk-shaped micro-resonators are studied intensively in MEMS-based inertial sensors since they offer significant advantages due to inherent symmetry in their geometries [1], including a high Q-factor, higher frequency, higher sensitivity, mode matching, good thermal stability, better resolution, and shock resistance in extreme conditions [2]. The disk-shaped resonators often suffer from small displacement amplitude and require precise fabrication [3]. The ring-shaped resonators have higher performance since they are more or less immune to the disturbances of the environment, such as random vibrations and temperature variations [4]. Additionally, since the advancement of deep reactive ion etch (DRIE)-based high-aspect ratio etching, the out-of-plane modes of vibration of the ring resonators are suppressed [5].

Ring resonators are widely preferred in MEMS Coriolis vibratory gyroscopes. Rotation of the vibrating ring activates the Coriolis force which exerts a tangential force. This tangential Coriolis force would generate a secondary degenerate sensing (complementary) mode of vibration of the ring, which is coupled with the primary mode. These two modes of vibration where nodal diameters are separated by *π*/4, are called degenerate modes of vibration since both have the same frequency due to the symmetry of geometry and material. These degenerate modes are also called drive and sense modes, respectively, and to maximize the energy transfer between these two modes, the resonator is usually driven in its resonant frequency [6].

By applying a time-varying voltage to the electrodes, the ring resonator vibrates. Accurately determining the working frequency of resonators is of paramount importance for operating it as a vibratory inertial sensor. The natural frequency of in-plane modes of vibration of a ring resonator depends on its material and geometrical parameters. However, when actuated electrically by sinusoidal actuation (AC), the working frequency is changed due to static (DC) bias voltage which causes the electrostatic softening of the stiffness, called electrical spring constant. Assuming a thin ring, the equations of motion of the degenerate modes of the ring resonators are simplified by neglecting in-plane inertia, transverse shear deformation, and rotary inertia [7,8]. If the wavelength of the vibration is larger than the thickness of the ring [9], thin ring assumption is valid. These equations are coupled due to rotation and electrostatic forces. Gallacher et al. [10] gave the equation of motions of a non-rotating ring resonator considering electrostatic force and neglecting stiffness and mass of the spokes in both radial and tangential directions. Sieberer et al. [11] gave the equation of motions of a non-rotating ring resonator considering electrostatic force and stiffness of the spokes in both radial and tangential directions and neglecting their mass. Gebrel et al. [12] considered the electrostatic force and the stiffness of the inside spokes in the radial direction only and neglected the mass and stiffness of spokes in the tangential direction of a non-rotating ring.

The equation of motion of a ring resonator in degenerate modes of vibration is nonlinear due to electrostatic force, damping, and large deflection. The solution characteristics, such as existence, uniqueness, and superposition, are not valid in nonlinear vibration [13], and resonant frequencies and mode shapes are amplitude-dependent [14] and exhibit non-exponential decay [15]. Therefore, most of the research works (Antonello et al. [6], Hu et al. [16], Yu et al. [17], Gallacher et al. [10], and Asokanthan and Cho [18]) have considered linear equations of motion. Most of them have considered the point evaluation or considered that ring beams, when they bend, will have the same distance between them and the electrode in the whole curvature; whereas, it is not as it bends that the centre will be way closer to the electrode than the edge of the electrode, so due to this there was a nonlinearity present. Evensen [9] was the first to study the nonlinearity due to the large deflection in the vibration of a thin non-rotating ring excited by a general harmonic loading. Liang et al. [4] considered Evensen [9] and proposed nonlinear radial and tangential displacement function and nonlinearity due to electrostatic force to derive the nonlinear coupled equation of motion. Sieberer et al. [11] considered electrostatics as the only source of nonlinearity. Nonlinearity due to the large deflection theory can be neglected considering the amplitude of vibration is less than the thickness of the ring.

The electrode arrangements greatly influence the dynamic performance of the ring resonators. This study’s main objective is to analyze the dynamic performance of a ring resonator with eight inside spokes in the form of twin circular curve beams (rose petals). Effective mass, effective stiffness, and applied electrostatic force were determined and compared with numerical results using ANSYS 2022 R1 software and experiment. In the next section, the theoretical background of the equations of motion was discussed, followed by the design of a ring resonator with eight rose petal spokes inside and eight electrodes outside. In Section 4, the calculation of effective mass and stiffness of the spokes, dynamic analysis of the designed ring resonator using the equation of motion, and numerical approach are given. In Section 5, the fabrication of the experimental method for the determination of resonance frequency is discussed, followed by conclusions in the Section 6.

## 2. Equations of Motion of a Vibrating Ring Resonator

### 2.1. Kinetic and Potential Energy of the Ring

In the case of small-amplitude vibration, it is expected that for a periodic excitation, the deformation of a shell/ring would be in the form of a standing wave and symmetric concerning the application point of load [7]. The radial displacement of a ring due to vibration can be expressed in the form of a trigonometric series as follows [19]:

(1) $$u_{r}=a_{n}\cos n\theta +b_{n}\sin n\theta$$

where $a_{n}$ and $b_{n}$ are the time-dependent amplitudes of the nth mode of vibration for the drive and sense modes, respectively. The equation is dependent on the nth variable which gives us the displacement based on the modes of the ring resonator. So, we can use the equation as the general one, but here we mainly concentrate on the n = 2. The figure of these degenerate modes are presented and explained in the Section 4.1. Where we can view the deformation of the ring resonator in Figure 4. Assuming pure bending of the ring without any stretching and using small deflection theory, the tangential component of displacement is determined as follows [20]:

(2) $$u_{\theta }=\frac{1}{n}\left(a_{n}\sin n\theta -b_{n}\cos n\theta \right)$$

Using Equations (1) and (2), the in-plane components of the velocity of any particle on the ring in the radial and tangential directions are determined as:

(3) $${\dot{u}}_{r}={\dot{a}}_{n}\cos n\theta +{\dot{b}}_{n}\sin n\theta$$

(4) $${\dot{u}}_{\theta }=\frac{1}{n}\left({\dot{a}}_{n}\sin n\theta -{\dot{b}}_{n}\cos n\theta \right)$$

The kinetic energy of the ring is calculated as follows [20]:

(5) $$T_{R}=\frac{1+n^{2}}{4n^{2}}M_{r}\left[{\left({\dot{a}}_{n}\right)}^{2}+{\left({\dot{b}}_{n}\right)}^{2}\right]$$

where $M_{r}$ is the total mass of the ring. The potential energy of the ring is expressed as follows [20]:

(6) $$V_{R}=\frac{\pi {\left(1-n^{2}\right)}^{2}}{2{\left(R_{r}\right)}^{3}}EI_{r}\left[{\left(a_{n}\right)}^{2}+{\left(b_{n}\right)}^{2}\right]$$

where *E* is Young’s modulus, $I_{r}$ is the moment of inertia of the ring cross-sectional area with respect to the axis *z*, and $R_{r}$ is the mean radius of the ring.

### 2.2. Kinetic and Potential Energy of the Spokes

**Figure 1:**
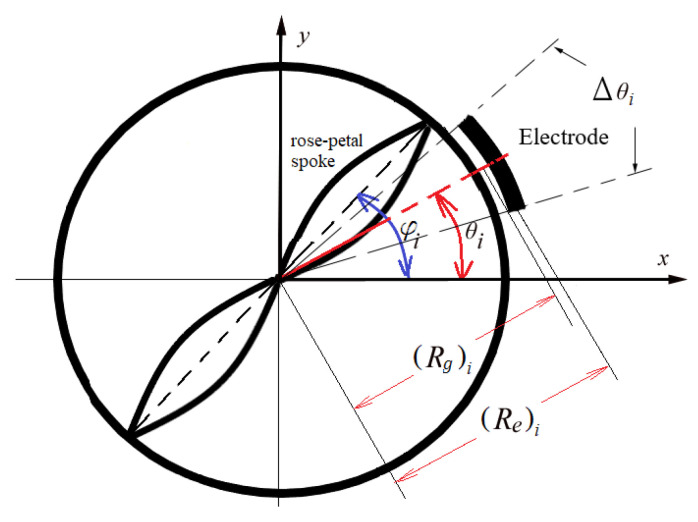
General arrangement and geometrical parameters of a ring resonator with electrodes positioned circumferentially outside and inside the spoke in the form of rose petals.

The effective mass of each spoke is considered as a lumped mass located at a point on the ring. Using the velocity of the ring, the kinetic energy of the spokes is calculated as follows:

(7) $$T_{S}=\frac{1}{2}m_{sr}\sum_{i=1}^{N_{s}}{{\left({\dot{u}}_{r}\right)}_{i}}^{2}+\frac{1}{2}m_{st}\sum_{i=1}^{N_{s}}{{\left({\dot{u}}_{\theta }\right)}_{i}}^{2}$$

where, $m_{sr}$ and $m_{st}$ are the effective masses of one spoke (rose petal) when it vibrates in the radial and tangential directions, respectively, and $N_{s}$ is the number of spokes. Substituting Equations (3) and (4) into Equation (7), the kinetic energy of the spokes would be:

(8) $$T_{s}=\frac{1}{2}m_{sr}\sum_{i=1}^{N_{s}}{\left[{\dot{a}}_{n}\cos n\varphi_{i}+{\dot{b}}_{n}\sin n\varphi_{i}\right]}^{2}+\frac{1}{2n}m_{st}\sum_{i=1}^{N_{s}}{\left[{\dot{a}}_{n}\sin n\varphi_{i}-{\dot{b}}_{n}\cos n\varphi_{i}\right]}^{2}$$

where $\varphi_{i}$ is the position angle of the *i*-th spoke with respect to *x-axis* (Figure 1). The potential energy of the spokes is determined as follows:

(9) $$V_{s}=\frac{1}{2}k_{sr}\sum_{i=1}^{N_{s}}{{\left(u_{r}\right)}_{i}}^{2}+\frac{1}{2}k_{st}\sum_{i=1}^{N_{s}}{{\left(u_{\theta }\right)}_{i}}^{2}$$

where $k_{sr}$ and $k_{st}$ are the stiffnesses of one spoke in the radial and tangential directions, respectively. Substituting Equations (1) and (2) into (9), the potential energy of the spokes would be:

(10) $$V_{s}=\frac{1}{2}k_{sr}\sum_{i=1}^{N_{s}}{\left(a_{n}\cos n\varphi_{i}+b_{n}\sin n\varphi_{i}\right)}^{2}+\frac{1}{2n}k_{st}\sum_{i=1}^{N_{s}}{\left[a_{n}\sin n\varphi_{i}-b_{n}\cos n\varphi_{i}\right]}^{2}$$

The Figure 1 shows the general representation of the rose petal designed resonator with its electrode position and the design variables associated with it.

### 2.3. Electrical Potential Energy of the Electrodes

Due to the applied voltage, an attractive force is imposed on the movable mass. A capacitor in the form of two parallel plates is generated, in which one plate is stationary and the other is free to move in the direction normal to the plane of the plates. The electrostatic potential energy of the capacitor is calculated based on the well-known parallel-plate approximation [21] as follows:

(11) $$U_{E}=\frac{1}{2}\varepsilon_{0}\varepsilon_{r}\frac{A_{o}}{d}\;V^{2}$$

where $\varepsilon_{0}=8.854\times 10^{-12}\;F/m$ is the permittivity of the free space between the plates in a vacuum, $\varepsilon_{r}$ is the permittivity of the medium between the plates, $A_{o}$ is the total overlap area of two plates in $m^{2}$, d is the gap distance between the plates in m, and V is the voltage applied to the electrode in volts. As the electrodes are positioned circumferentially outside of the ring, the overlap area is the length of the electrode times the height of the ring (Figure 1), as follows:

(12) $$A_{o}=\sum_{i=1}^{{\left(N_{e}\right)}_{j}}{\left(h_{r}R_{g}\Delta \theta \right)}_{i}$$

where ${\left(N_{e}\right)}_{j}$ is the number of electrodes in one group with the same applied voltage, ${\left(h_{r}\right)}_{i}$ is the thickness of each electrode in one group, ${\left(R_{g}\right)}_{i}$ is the mean radius of the gap between each electrode and outside ring in one group of electrodes, and $\Delta \theta_{i}$ is the central angle in front of each electrode in one group of electrodes (Figure 1).

Depending on the type of the electrode and method of detection, amplitude modulation (AM) or frequency modulation (FM), the applied voltage could be DC bias voltage and/or an alternative (AC) voltage as:

(13) $$\left\{\begin{array}{c} V=V_{DC}\pm V_{AC}\sin\omega t;\;\mathrm{Drive}\;\mathrm{Electrodes}\;\mathrm{and}\;\mathrm{Sense}\;\mathrm{Electrodes}\;\left(\mathrm{FM}\right)\\ V=V_{DC};\;\mathrm{Sense}\;\mathrm{Electrodes}\;\left(\mathrm{AM}\right) \end{array}\right.$$

The distance between electrodes and the ring changes due to the applied electrostatic force, thus Equation (11) for one electrode is modified as follows:

(14) $${\left(U_{E}\right)}_{j}={\left[{\left(V_{DC}\right)}_{j}\pm {\left(V_{AC}\right)}_{j}\sin\omega t\right]}^{2}\sum_{i=1}^{{\left(N_{e}\right)}_{j}}\frac{1}{2}\varepsilon_{0}\varepsilon_{r}{\left(\frac{h_{r}R_{g}\Delta \theta }{d-u_{r}}\right)}_{i}$$

Using Taylor’s expansion, and assuming the radial displacement of the ring due to vibration is small in comparison to gap distance, i.e., $u_{r}\ll d$, [1], Equation (14) is expanded to fourth-order approximation as follows [11]:

$${\left(U_{E}\right)}_{j}={\left[{\left(V_{DC}\right)}_{j}\pm {\left(V_{AC}\right)}_{j}\sin\omega t\right]}^{2}\overset{{\left(N_{e}\right)}_{j}}{\underset{i=1}{\sum }}\frac{1}{2}\varepsilon_{0}\varepsilon_{r}\;{\left(\frac{h_{r}R_{g}\Delta \theta }{d}\right)}_{i}\left[1+{\left(\frac{u_{r}}{d}\right)}_{i}+{\left(\frac{u_{r}}{d}\right)}_{i}^{2}+{\left(\frac{u_{r}}{d}\right)}_{i}^{3}\right]$$

Since all electrodes have the same thickness, radius, central angle, and air gap distance, total electrical potential energy is expressed as:

(15) $$U_{E}=\frac{1}{2}\varepsilon_{0}\varepsilon_{r}\frac{h_{r}R_{g}}{d}\sum_{j=1}^{N_{G}}{\left[{\left(V_{DC}\right)}_{j}\pm {\left(V_{AC}\right)}_{j}\sin\omega t\right]}^{2}\sum_{i=1}^{{\left(N_{e}\right)}_{j}}\int_{\theta_{i}-\frac{\Delta \theta }{2}}^{\theta_{i}+\frac{\Delta \theta }{2}}\left[1+\frac{u_{r}}{d}+{\left(\frac{u_{r}}{d}\right)}^{2}+{\left(\frac{u_{r}}{d}\right)}^{3}\right]d\theta \;$$

where $N_{G}$ is the total number of groups of electrodes.

### 2.4. Equation of Motion

Having the kinetic and potential energy of a vibrating ring, it is possible to drive the equation of motion using the well-known Lagrange’s equation [22] as follows:

(16) $$\left\{\begin{array}{c} \frac{d}{dt}\left[\frac{\partial }{\partial {\dot{a}}_{n}}\left(T_{R}+T_{S}\right)\right]-\frac{\partial }{\partial a_{n}}\left(T_{R}+T_{S}\right)+\frac{\partial }{\partial a_{n}}\left(V_{R}+V_{S}-U_{E}\right)=\frac{\partial }{\partial {\dot{a}}_{n}}\left(W_{D}\right)\\ \frac{d}{dt}\left[\frac{\partial }{\partial {\dot{b}}_{n}}\left(T_{R}+T_{S}\right)\right]-\frac{\partial }{\partial b_{n}}\left(T_{R}+T_{S}\right)+\frac{\partial }{\partial b_{n}}\left(V_{R}+V_{S}-U_{E}\right)=\frac{\partial }{\partial {\dot{b}}_{n}}\left(W_{D}\right) \end{array}\right.$$

where $W_{D}$ is the energy lost due to damping [5]. Neglecting damping and nonlinear terms of the electrostatic force, and introducing the following non-dimensional variables and parameters,

$$a_{n}^{*}=\frac{a_{n}}{R_{r}}\;b_{n}^{*}=\frac{b_{n}}{R_{r}}\;w_{s}^{*}=\frac{w_{s}}{R_{r}}\;w_{r}^{*}=\frac{w_{r}}{R_{r}}\;h_{s}^{*}=\frac{h_{s}}{R_{r}}\;h_{r}^{*}=\frac{h_{r}}{R_{r}}\;R^{*}=\frac{R_{s}}{R_{r}}\;d^{*}=\frac{d}{R_{r}}\;R_{g}^{*}=\frac{R_{g}}{R_{r}}\;m_{sr}^{*}=\frac{1}{M_{r}}m_{sr}\;m_{st}^{*}=\frac{1}{M_{r}}m_{st}\;\tau^{2}=\frac{1}{M_{r}}\frac{\pi EI_{r}}{{\left(R_{r}\right)}^{3}}\;\omega_{N}^{*}=\frac{1}{\tau }\omega_{N}\;\omega_{AC}^{*}=\frac{1}{\tau }\omega_{AC}\;t^{*}=t\times \tau \;k_{sr}^{*}=\frac{{\left(R_{r}\right)}^{3}}{\pi EI_{r}}k_{sr}\;k_{st}^{*}=\frac{{\left(R_{r}\right)}^{3}}{\pi EI_{r}}k_{st}\;{\left(V^{*}\right)}^{2}=\frac{1}{\tau^{2}}\frac{\varepsilon_{0}\varepsilon_{r}h_{r}R_{g}}{2M_{r}d^{3}}V^{2}$$

the equations of motion of two degrees of freedom are determined as follows:

(17) $$m^{*}\left[\begin{array}{c} {\ddot{a}}_{n}^{*}\\ {\ddot{b}}_{n}^{*} \end{array}\right]+\tau^{2}\left[k^{*}-\sum_{j=1}^{N_{G}}{\left(V_{j}^{*}\right)}^{2}{\left(k_{E}^{*}\right)}_{j}\right]\left[\begin{array}{c} a_{n}^{*}\\ b_{n}^{*} \end{array}\right]=\tau^{2}\sum_{j=1}^{N_{G}}{\left(V_{j}^{*}\right)}^{2}{\left(f_{E}^{*}\right)}_{j}$$

where $m^{*}$, $k^{*}$, ${\left(k_{E}^{*}\right)}_{j}$, and ${\left(f_{E}^{*}\right)}_{j}$, are the positive non-dimensional mass matrix, the non-dimensional positive stiffness matrix, the electrical stiffness matrix of the *j*-th electrode, and the non-dimensional force vector, respectively, and are expressed as follows:

$$m^{*}=\frac{1+n^{2}}{2n^{2}}\left[\begin{array}{cc} 1 & 0\\ 0 & 1 \end{array}\right]+m_{sr}^{*}\left[\begin{array}{cc} \overset{N_{s}}{\underset{i=1}{\sum }}\cos^{2}n\varphi_{i} & \frac{1}{2}\overset{N_{s}}{\underset{i=1}{\sum }}\sin2n\varphi_{i}\\ \frac{1}{2}\overset{N_{s}}{\underset{i=1}{\sum }}\sin2n\varphi_{i} & \overset{N_{s}}{\underset{i=1}{\sum }}\sin^{2}n\varphi_{i} \end{array}\right]\;+\frac{1}{n^{2}}m_{st}^{*}\left[\begin{array}{cc} \overset{N_{s}}{\underset{i=1}{\sum }}\sin^{2}n\varphi_{i} & -\frac{1}{2}\overset{N_{s}}{\underset{i=1}{\sum }}\sin2n\varphi_{i}\\ -\frac{1}{2}\overset{N_{s}}{\underset{i=1}{\sum }}\sin2n\varphi_{i} & \overset{N_{s}}{\underset{i=1}{\sum }}\cos^{2}n\varphi_{i} \end{array}\right]\;k^{*}={\left(1-n^{2}\right)}^{2}\left[\begin{array}{cc} 1 & 0\\ 0 & 1 \end{array}\right]+k_{sr}^{*}\left[\begin{array}{cc} \overset{N_{s}}{\underset{i=1}{\sum }}\cos^{2}n\varphi_{i} & \frac{1}{2}\overset{N_{s}}{\underset{i=1}{\sum }}\sin2n\varphi_{i}\\ \frac{1}{2}\overset{N_{s}}{\underset{i=1}{\sum }}\sin2n\varphi_{i} & \overset{N_{s}}{\underset{i=1}{\sum }}\sin^{2}n\varphi_{i} \end{array}\right]\;+\frac{1}{n^{2}}k_{st}^{*}\left[\begin{array}{cc} \overset{N_{s}}{\underset{i=1}{\sum }}\sin^{2}n\varphi_{i} & -\frac{1}{2}\overset{N_{s}}{\underset{i=1}{\sum }}\sin2n\varphi_{i}\\ -\frac{1}{2}\overset{N_{s}}{\underset{i=1}{\sum }}\sin2n\varphi_{i} & \overset{N_{s}}{\underset{i=1}{\sum }}\cos^{2}n\varphi_{i} \end{array}\right]\;{\left(k_{E}^{*}\right)}_{j}=-{\left(N_{e}\right)}_{j}\;\Delta \theta \left[\begin{array}{cc} 1 & 0\\ 0 & 1 \end{array}\right]+\frac{1}{2n}\;\left[\begin{array}{cc} \overset{{\left(N_{e}\right)}_{j}}{\underset{i=1}{\sum }}-\left[\sin2n\left(\theta_{i}+\frac{\Delta \theta }{2}\right)-\sin2n\left(\theta_{i}-\frac{\Delta \theta }{2}\right)\right]\; & \overset{{\left(N_{e}\right)}_{j}}{\underset{i=1}{\sum }}\left[\cos2n\left(\theta_{i}+\frac{\Delta \theta }{2}\right)-\cos2n\left(\theta_{i}-\frac{\Delta \theta }{2}\right)\right]\\ \overset{{\left(N_{e}\right)}_{j}}{\underset{i=1}{\sum }}\left[\cos2n\left(\theta_{i}+\frac{\Delta \theta }{2}\right)-\cos2n\left(\theta_{i}-\frac{\Delta \theta }{2}\right)\right] & \overset{{\left(N_{e}\right)}_{j}}{\underset{i=1}{\sum }}\left[\sin2n\left(\theta_{i}+\frac{\Delta \theta }{2}\right)-\sin2n\left(\theta_{i}-\frac{\Delta \theta }{2}\right)\right]\; \end{array}\right]\;{\left(f_{E}^{*}\right)}_{j}=\frac{1}{n}d^{*}\left[\begin{array}{c} \overset{{\left(N_{E}\right)}_{j}}{\underset{i=1}{\sum }}-\left[\sin n\left(\theta_{i}+\frac{\Delta \theta }{2}\right)-\sin n\left(\theta_{i}-\frac{\Delta \theta }{2}\right)\right]\;\\ \overset{{\left(N_{E}\right)}_{j}}{\underset{i=1}{\sum }}\left[\cos n\left(\theta_{i}+\frac{\Delta \theta }{2}\right)-\cos n\left(\theta_{i}-\frac{\Delta \theta }{2}\right)\right]\; \end{array}\right]$$

## 3. Design of the Ring Resonator

**Figure 2:**
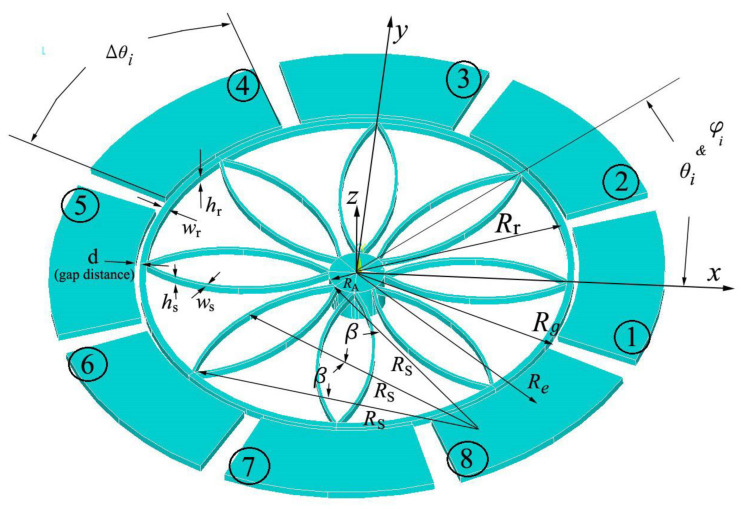
The geometry and definition of the geometrical parameters of the ring resonator considered in this study.

To show the applicability of Equation (17) and examine the effect of the electrostatic force on the dynamic behavior of the ring resonators, a ring with eight spokes and eight electrodes is designed and analyzed (Figure 2). The electrodes are positioned circumferentially outside the ring in the form of an arc with a central angle of Δθ. The center of the electrodes is located at the position of the spokes ($\theta_{i}$ = $\varphi_{i}$) to have maximum deflection of the spokes which results in the maximum elastic force.

In the Figure 2, 2β and $R_{S}$ are the central angle and the radius of each circular curve beam in the plane of the ring, respectively. Table 1 depicts the geometrical characteristics of the designed ring.

## 4. Dynamic Analysis of the Designed Ring Resonator

As Table 1 shows the geometrical parameters of the silicon-based ring resonator, some of the main parameters that need to be considered are the $R_{A}$, $w_{S}$, $w_{r}$, and d. Here, the d plays a prominent role in the dynamic analysis due to the electrostatic force dependence on that parameter and how it is only limited by the fabrication capability. The ring resonator has eight identical spokes positioned symmetrically inside the ring, and the matrices of the equation of motion for the n = 2 mode of vibration would simplify as follows:

$$m^{*}=\left(\frac{5}{8}+4m_{sr}^{*}+m_{st}^{*}\right)\left[\begin{array}{cc} 1 & 0\\ 0 & 1 \end{array}\right]\;k^{*}=\left(9+4k_{sr}^{*}+k_{st}^{*}\right)\left[\begin{array}{cc} 1 & 0\\ 0 & 1 \end{array}\right]$$

The electrical stiffness matrix for electrodes No. 1 and 5 in Figure 2 is determined as follows:

$${\left(k_{E}^{*}\right)}_{15}=\left[\begin{array}{cc} 2.3811 & 0\\ 0 & 0.4115 \end{array}\right]$$

### 4.1. Natural Frequency of the Ring Resonator

Neglecting the stiffness softening due to electrical force, $k_{E}^{*}$, the natural frequency of a ring resonator, is calculated using the following eigenvalue equation:

(18) $$\|k^{*}-{\left(\omega^{*}\right)}^{2}m^{*}\|=0$$

The equation for the calculation of the non-dimensional frequency of the ring with eight spokes in the n = 2 mode of vibration would be as follows:

(19) $${\left(\omega_{2}^{*}\right)}^{2}=\frac{\;9+4k_{sr}^{*}+k_{st}^{*}}{\frac{5}{8}+4m_{sr}^{*}+m_{st}^{*}}$$

The calculation of spoke stiffness is an essential step in determining natural frequency. Rahbar Ranji et al. [23] gave equations for the calculation of the stiffness of folded beams. For any curved beam fixed at one end and free to displace at the other end, Rahbar Ranji et al. [20] gave the following equation for the calculation of stiffness:

(20) EIsRs3ks=β+12sin2βcos2α−2βcos2α sin2β−12sin2β−1βsin2β2β−12sin2βcos2α−2βsin2αsin2β sin2α2

where α is the direction that the beam is free to displace at the free end, E is Young’s modulus, and $I_{s}$ is the moment inertia of the circular curve beam with respect to the *z*-axis. Substituting α by 0 and 90 degrees, $\frac{1}{2}k_{sr}^{*}$, and $\frac{1}{2}k_{st}^{*}$, the stiffness of one circular curve beam (half-spoke) in radial and tangential directions are determined, respectively (Table 2). To calculate the effective mass of one circular curve beam in radial and tangential directions, the finite element method (FEM) is used, and the natural frequencies of a curved beam fixed at one end and free to displace at the other end are determined. Having natural frequencies and stiffness, the effective mass is determined (Table 2).

**Figure 3:**
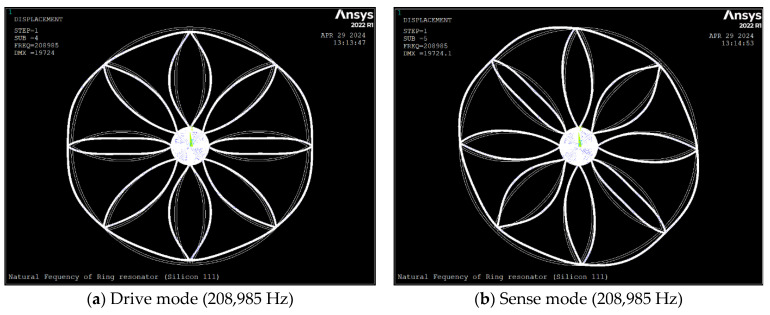
Natural frequency in *n* = 2 mode of vibration of the ring (silicon [111]). The brighter color showing the deformed mode shape and the lighter showing undeformed ring resonator.

**Figure 4:**
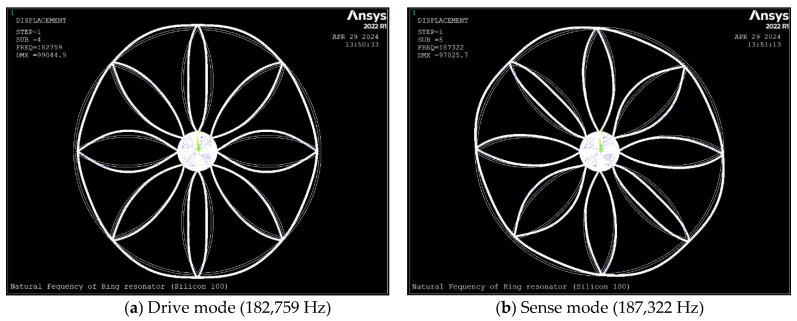
Natural frequency in *n* = 2 mode of vibration of the ring (silicon [100]). The brighter color showing the deformed mode shape and the lighter showing undeformed ring resonator.

Figure 3 and Figure 4 depict *n* = 2 mode shapes and natural frequencies of the ring which were obtained using computer code ANSYS [24]; as MEMS devices are fabricated using anisotropic silicon material, we have considered silicon [111] and [100] as device materials. The PLANE183 element with a fine mesh size is used as a meshing. The ring resonator is suspended, and it is anchored to the centre. We have the radial and tangential displacements of all nodes on the perimeter of the anchor fixed as the boundary conditions.

The Figure 3 and Figure 4 shows the natural frequency and the degenerate mode shapes for the n = 2 mode of vibration of the ring for silicon [111] and [100]. Table 3 depicts the non-dimensional frequencies calculated by FEM and compared with Equation (19). As seen, using anisotropic silicon [100] induced about a 2.4% frequency split, where drive and sense frequencies increased by about 4.8% and 7.4%, respectively. The FE results show that the mode shape of the ring is a combination of the general ring vibration and the local vibration of the ring between spokes which occurs due to the low stiffness of the ring. Additionally, comparing the results of FEM and Equation (19) reveals a deviation of 13.2%. This is partially caused by the ring’s local vibration between the spokes, which the analytical method ignores.

### 4.2. Pull-In Voltage Analysis

The applied force due to electrostatic deforms the ring and activates the elastic forces of the spokes. When the bias DC voltage reaches a certain critical value, the electrostatic force surpasses the induced elastic force, making the ring incapable of maintaining its equilibrium state. This voltage is called pull-in voltage, and the corresponding displacement of the ring is called pull-in displacement. Static or dynamic analysis is used for the determination of pull-in voltage [25,26,27]. Using parallel-plate theory and static equilibrium, the pull-in voltage is determined as [28]:

(21) $$V=\sqrt{\frac{8\cdot k\cdot d^{3}}{27\cdot \varepsilon_{0}\cdot \varepsilon_{r}\cdot A_{o}}}$$

where k is the elastic stiffness. Pull-in displacement is determined as $u_{PI}=0.333d$. Using non-dimensional parameters, Equation (21) for the ring is expressed as follows:

(22) $$V_{PI}^{*}=2\sqrt{\frac{9+4k_{sr}^{*}+k_{st}^{*}}{27\cdot \Delta \theta \cdot N_{E}}}$$

Substituting the geometrical and material properties of the ring in the case electrodes No. 1 and 5 are activated, the non-dimensional pull-in voltage is calculated as 14.80. Applying the static equilibrium of the ring, the non-dimensional pull-in voltage is calculated by equating elastic force and electrostatic force as follows:

(23) $$k^{*}a_{n}^{*}-\left[{\left(k_{E}^{*}\right)}_{j}a_{n}^{*}+{\left(d_{1}^{*}\right)}_{j}{\left(a_{n}^{*}\right)}^{2}+{\left(f_{E}^{*}\right)}_{j}\right]\cdot N_{E}\cdot {\left(V^{*}\right)}^{2}=0$$

Equations (A3)–(A6) are used to calculate electrostatic force including nonlinear terms. 

**Figure 5:**
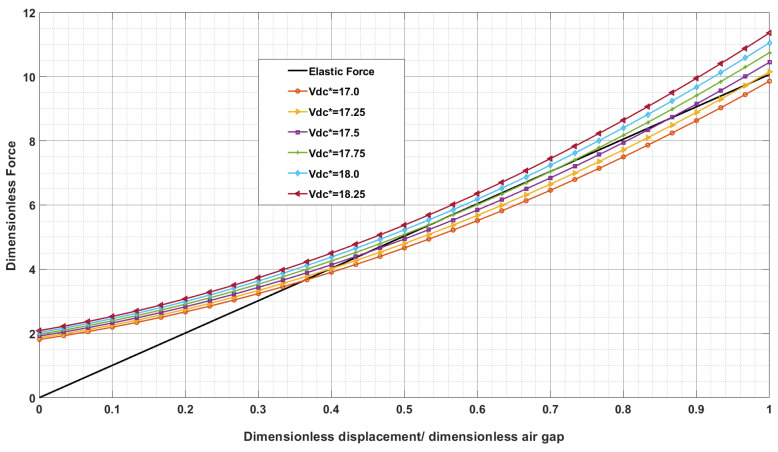
Elastic and electrostatic force versus displacement of the ring in drive coordinate due to DC bias voltage applied to electrodes number 1 and 5 (silicon [111]).

Figure 5 depicts non-dimensional elastic and electrostatic forces as a function of non-dimensional displacement in drive coordinates for different values of non-dimensional applied voltage. Here the pull-in voltage is determined for the different dimensionless air gaps when the elastic force line acts as a tangential line to the curves of different voltages. The tangential point of the curve at the dimensionless air gap with a voltage corresponds to the pull-in voltage. 

**Figure 6:**
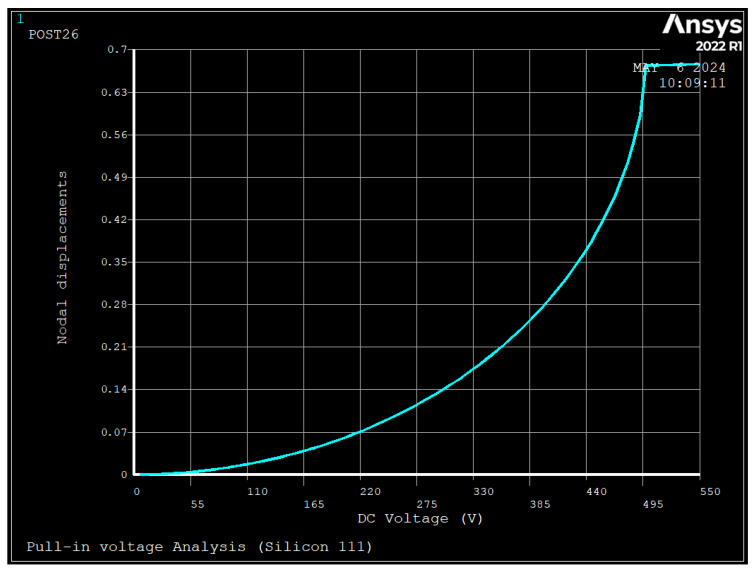
FE results of drive coordinate displacement versus applied voltage to electrodes number 1 and 5 (V_PI_ = 504 V, u_PI_ = 0.67 um, Silicon [111]).

**Figure 7:**
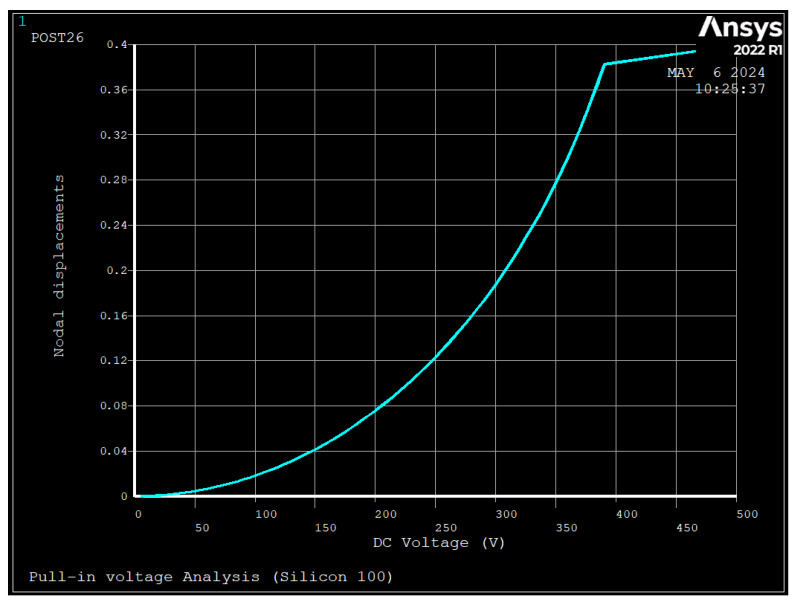
FE results of drive coordinate displacement versus applied voltage to electrodes number 1 and 5 (V_PI_ = 392 V, u_PI_ = 0.39 um, Silicon [100]).

Figure 6 and Figure 7 depict the results of FEM for calculation of pull-in voltage, and Table 4 depicts the non-dimensional pull-in voltage and pull-in displacement calculated by FEM and compared with Equation (23). The figure shows the displacement of the nodes as the DC voltage increased, and at one point, instead of increasing the displacement, became constant, which shows the pull-in voltage as the device collapsed at that voltage point.

As seen, the deviations between the results of FEM and Equations (22) and (23) are 33.8% and 60%, respectively. These deviations are due to the assumption made in parallel-plate theory where: two plates remain parallel after deformation, the lumped mass model neglects the coupling of structure and electrostatic [27], there is a reduction in the effective area due to deflection of the ring [29], and concentrated electrostatic force is applied at the position of spokes. Additionally, compared to silicon [100], silicon [111] had a 6.6% and 41% higher pull-in voltage and pull-in displacement, respectively.

### 4.3. Resonance Frequency Shift

The natural frequency of the ring resonator shifts due to applied bias DC voltage, called an electrical spring softening-induced frequency shift. To study the effect of bias DC voltage on the natural frequency, stiffness matrix, and electrical stiffness matrix due to applied DC voltage, these elements are determined and substituted in the following equation:

(24) $$\|k^{*}-\sum_{j=1}^{N_{G}}V_{j}^{2}{\left(k_{E}^{*}\right)}_{j}-{\left(\omega_{2}^{*}\right)}^{2}m^{*}\|=0$$

To validate the results of Equation (24), FEM is used to calculate the shift of resonance frequency for the ring using silicon [111] and [100]. Electrostatic analysis using a PLANE183 element for ring and spokes, and PLANE223 for air gap are used.

**Figure 8:**
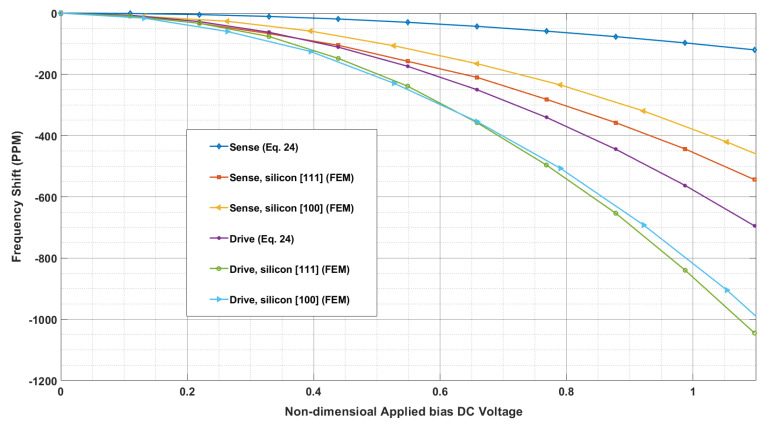
Resonance frequencies shift as a function of bias DC voltages applied to electrodes No. 1 and 5. Eq. 24 refereeing the analytical equation no 24.

Figure 8 depicts the variation in resonance frequencies of the ring resonator as a function of non-dimensional bias DC voltage applied to electrodes numbers 1 and 5, calculated using Equation (24) and FEM with silicon [111] and [100]. As we mentioned earlier, the frequency shifts due to the high DC bias which creates the electrical spring constant due to the electrostatic softening of the stiffness. As seen, both methods have the same tendency, and by increasing bias DC voltage, the shift of resonance frequency increases. The rate of shift of frequency increases by increasing bias DC voltage and the deviation between analytical approach and numerical method increases by increasing applied voltage.

### 4.4. Harmonic Analysis of the Ring

**Figure 9:**
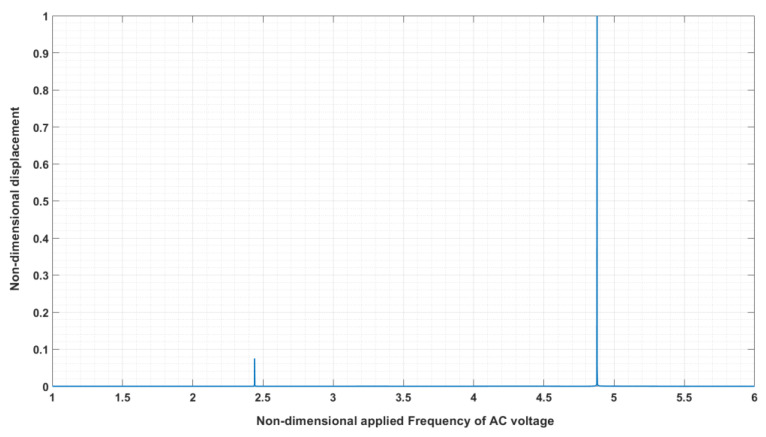
Harmonic response of the ring due to electrostatic force applied to electrodes No. 1 and 5, using Equation (17) (silicon [111]).

As mentioned, to have maximum energy transfer from drive mode of vibration to sense mode due to rotation, the resonator is excited in resonance frequency. To determine the resonance frequency of the ring resonator, a non-dimensional DC bias voltage of $4.55E\left(-3\right)$ and AC of $4.55E\left(-4\right)$ is applied to electrodes No. 1 and 5. Equation (17) is used to determine the harmonic response (Figure 9). So, the figure depicts the sweep of frequency of the AC voltage versus the displacement obtained at electrodes 1 and 5, which is where the *n* = 2 drive mode will have maximum displacement.

**Figure 10:**
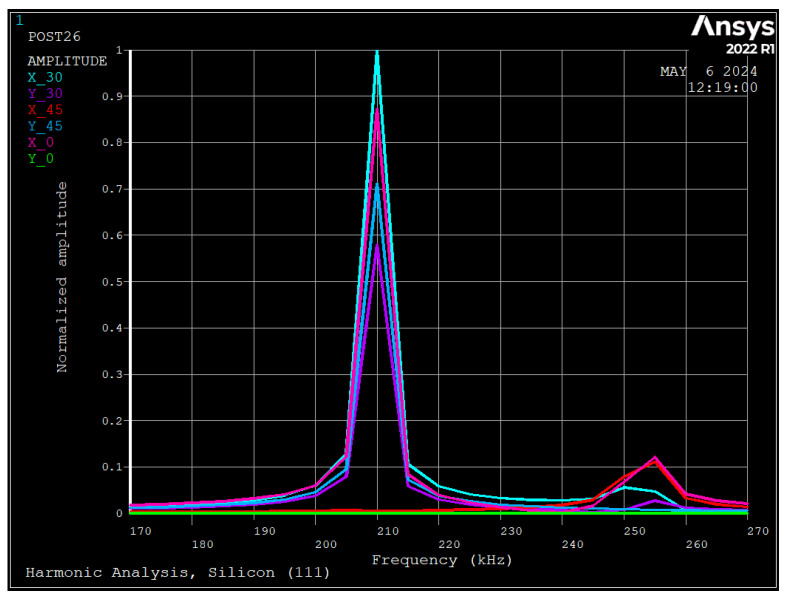
Harmonic response due to applied voltage using FEM (Silicon [111]).

As seen, the non-dimensional resonance frequency is 4.88 which matches the natural frequency shown in Table 3, since at this non-dimensional frequency, the frequency shift is almost zero (Figure 8). There is a vibration at the non-dimensional frequency of 2.4 with a very low amplitude, which corresponds to the rocking mode of vibration. Figure 10 depicts FEM results at radial and tangential displacements at different electrode positions for harmonic analysis of the ring with material silicon [111].

As seen, the normalized radial displacements at θ= 0, 30, and 45 degrees are 0.88, 1.0, and 0.0, respectively. Thus, since the maximum displacement occurs at θ= 30 degrees, it indicates that local vibration of the ring between spokes has occurred in addition to general vibration, which also was observed in Figure 3a. The tangential displacements at θ= 0, 30, and 45 degrees are 0.0, 0.58, and 0.70, respectively. Thus, the local vibration of the ring between spokes causes tangential displacement of the ring at 30 and 45 degrees. As mentioned in Section 4.2, the deviation between the analytical approach and FEM is about 13.2%, due to local vibration of the ring.

## 5. Fabrication and Testing of Ring Resonator

### 5.1. Fabrication of the Ring Resonator

Silicon is the material that is utilized to fabricate MEMS, and its mechanical properties depend on crystal orientation. The impact of silicon crystal orientation extends beyond just Young’s modulus. Eley et al. [30] studied the material properties of silicon in different orientations. Silicon [111] is an isotropic material with Young’s modulus of 188.43 GPa, and silicon [100] is a cubic material with Young’s modulus in x and y directions equal to 130.82 GPa. Ghaffari et al. [31] have shown that the quality factor (Q) of MEMS resonators can vary significantly with crystal orientation, noting up to a 20% difference depending on whether the orientation is <100>, <110>, or <111>. This variation is critical because the Q factor directly influences the resonator’s performance by affecting its energy dissipation rates. Additionally, frequency splitting in flexural disk resonators, caused by the anisotropic nature of silicon, is another performance aspect. The finite-element modelling aligns with experimental observations, indicating that even polysilicon, which is generally considered isotropic, can exhibit significant performance variations due to underlying crystal properties. Silicon [100] is a widely used material for the fabrication of MEMS resonators [32] due to accuracy and low cost of fabrication; however, anisotropy in the stiffness generates a frequency split [33].

**Figure 11:**
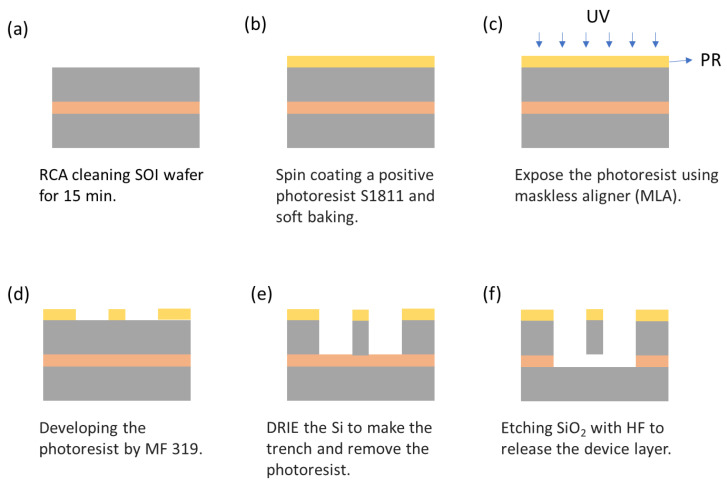
Fabrication process flow diagram of SOI-based resonator.

**Figure 12:**
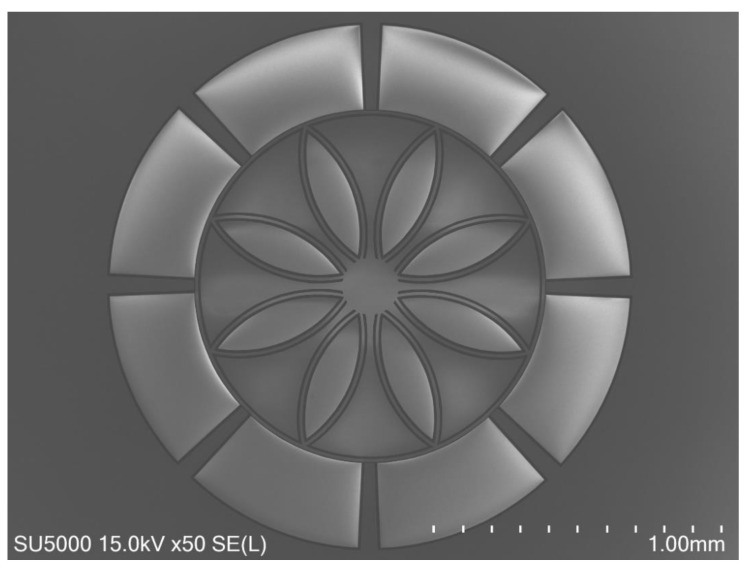
Scanning electron microscopy (SEM) image showing of the fabricated ring resonator with twin circular curve (petal) shaped inner spring support and surrounding electrodes.

A p-type 100 mm diameter silicon-on-insulator (SOI) wafer (Ultrasil LLC, Hayward, USA) with a device layer of 25 μm (0.001–0.003 Ω⋅cm), SiO_2_ box layer of 5 μm, and handle layer of 500 μm (0.005–0.02 Ω⋅cm) was used as starting material. The wafer was first cleaned through an RCA (Radio Corporation of America, New York, USA) process (Figure 11a). Then, Shipley S1811 photoresist was applied to the wafer via spin-coating at 2000 rpm using a Brewer Science CEE 200X spinner. The wafer then underwent a soft bake at 120 °C for 2 min on a Brewer Science 1300X vacuum hotplate (Figure 11b). Its pattern was exposed using MLA (MLA-150, Heidelberg Instruments, Heidelberg, Germany), with UV laser set to 405 nm, exposure at 125 mJ/cm^2^ dose, and -2 defocus, and developed in MF-319 developer for 45 s (Figure 11c,d). Then, the pattern was transferred into the SOI substrate by the deep reactive ion etching (DRIE) technique using Plasmalab system 100 ICP380 (Oxford Instruments, Abingdon, United Kingdom) with a high-density 380 mm ICP Source (2 MHz, 3 kW). The DRIE experiment was performed by using the Bosch process with an etching (160 sccm SF6 and 160 sccm C4F8 flow rates) cycle RF power of 1000 W. During the etching step, SF_6_ plasma isotropically etches silicon and would only be allowed to etch for a limited depth, which is usually less than one micron, while the C_4_F_8_ step deposits a passivation layer to protect the sidewalls, thus achieving anisotropic etching. The Bosch deep and etching cycle times were kept at 5 s and 7 s, respectively (Figure 11e). To minimize the notching effect, high-frequency (13.56 MHz) etching was applied for 100 cycles, followed by low-frequency (350 kHz) etching for the final 10 cycles, a critical adjustment that significantly reduced the charge accumulation and consequent deflection of etching ions, effectively reduce notching. The etching depth was monitored using a profilometer (DektakXT, Bruker, Billerica, USA) to ensure consistent results across different DRIEs.. Subsequently, the photoresist was removed ultrasonically in acetone for 5 min. Then, it was immersed in IPA for 5 min. Finally, the box layer was etched with 49% hydrofluoric acid (HF) for 10 min to release the device layer (Figure 11f). The final device was packaged by wire bonding (West-Bond Inc., model 4546E). Figure 12 depicts the SEM of the fabricated ring resonator.

### 5.2. Testing of the Fabricated Ring

**Figure 13:**
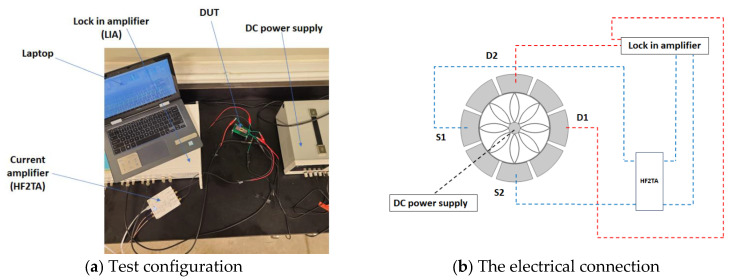
The image and schematic diagram showing the test setup for frequency response testing of the ring resonance for a forced vibration.

To determine how the resonator responds to electrical actuation and modulation, its resonance frequency is measured using the lock-in-amplifier (LIA). The test configuration is displayed in Figure 13a. Figure 13b depicts the electrical connection between the DC power source, LIA, and ring. To preserve the electrical polarity between the electrodes and the ring resonator, a DC power source is attached to the ring anchor and provides a DC voltage of 1.0 V. The AC voltage of 10 mV with 0 deg phase is applied to Electrode 1 and 10 mV with 180 deg phase to Electrode 3. As shown in the previous model section, the drive mode of the ring resonator is elliptical in shape. So, we are forced, with a small electrostatic force, to vibrate the device in the drive mode. We can accomplish this by adding the electrostatic force to the anti-nodal points which are Electrodes 1, 3, 5 and 7 as seen in Figure 2. Here, Electrode 1 and 5 and Electrode 3 and 7 are in pairs to form the elliptical shape. The response, represented by floating potentials, is obtained from the electrodes (S1 and S2) across from each other. The small displacement causes the sensed signal to typically be in the lower range, necessitating the use of a current amplifier. Thus, the output signal is amplified before it reaches the LIA with the aid of a current amplifier (HFT2A). The mechanically modulated signal in the ring that was brought about by resonance is demodulated using the respective drive signal within the LIA to eliminate noise.

**Figure 14:**
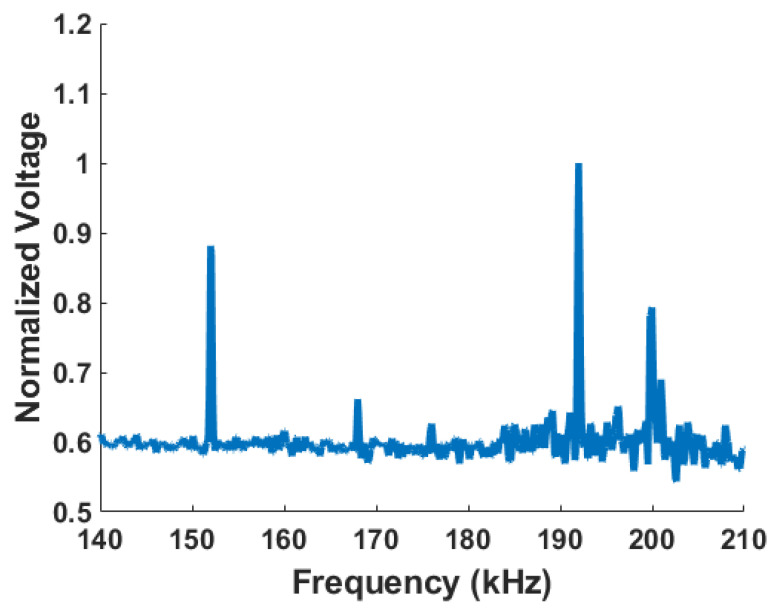
Test result of the ring resonator showing the frequency response and important frequencies (silicon [100]).

Here, we have used the LIA to perform a frequency sweep which is used to determine the resonance of the frequency. As the electrodes D1 and D2 are used to drive and S1 and S2 are used to sense, the *n* = 2 mode should have a large displacement. The frequency sweep is performed from 120 kHz to 220 kHz to determine the device’s resonance frequencies (Figure 14). Using the average of the initial values as a reference, the response has been shown as a frequency against the normalized voltage. The drive mode of *n* = 2 was presented at 192 kHz and in the rocking mode at 152 kHz.

**Figure 15:**
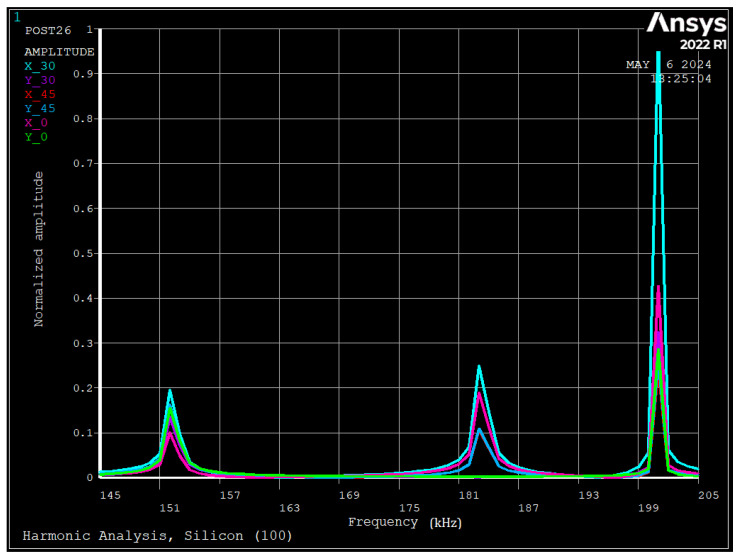
Simulated frequency response of the ring showing important frequencies (silicon [100]).

Figure 15 depicts the FE results for harmonic analysis of the ring with silicon [100], due to applied voltages of 1.0 V DC and 0.1 mV AC. Here we have obtained multiple peaks and all of them correspond to different modes, and we can differentiate them by the radial and tangential displacements in different electrode positions. The first peak is at 152 kHz, where the displacement of all points in radial and tangential directions are in the range of 0.1 and 0.2. Since all displacements are almost the same, it can be concluded that it is the rocking mode and matches well with the experiments (Figure 14). The second resonance frequency occurs at 183 kHz, where radial displacements at 0, 30, and 45 degrees are 0.20, 0.25, and 0.11, and tangential displacements are 0.0, 0.0, and 0.11, respectively. Since tangential displacement at 0 degrees is zero, it is *n* = 2 vibration; however, as maximum displacement occurs at 30 degrees, the mode shape is the interaction of local and global vibration. Compared with the experiment results, it reveals a 5% deviation that could be due to damping or fabrication errors. Since both analytical and experimental approaches have considered local and global vibration, the deviation is reduced from 13.2% to 5%. The third resonance occurs at 201 kHz which matches well with the experiment. The radial displacements at 0, 30, and 45 degrees are 0.42, 1.0, and 0.24, respectively. Since after this frequency, all tangential displacements are opposite to zero, it is the interaction of vibration and torsional modes.

Our devices can be used in practical applications for various industries. In telecommunications, MEMS resonators with the characteristics observed in our study can be utilized in RF filters and oscillators, improving the performance and reliability of communication devices. The high sensitivity and frequency tuning capabilities of the resonators make them ideal for precise signal processing. In the field of sensors, our MEMS resonators can enhance the performance of gyroscopes used in automotive and consumer electronics. The accuracy and stability observed in the resonance frequencies make these devices more reliable for motion detection and measurement. Also in the future, we want to include the damping in the analysis to see how much the squeeze and the slide damping affect the performance of the device.

## 6. Conclusions

The dynamic behavior of the ring resonator is important for the proper design of MEMS. The kinetic and potential energy of the ring and spokes were derived using the kinetic and potential energy analyses. We have formulated the equation which can be used to calculate the electrical potential energy of electrodes for any number of inside/outside circumferential electrodes. The circular curve beam’s (spokes’) effective mass and stiffness were computed. The well-known Lagrange’s method was used to derive the equations of motion for a ring acting under the influence of electrostatic force and we have neglected the damping here. These equations were used to study the natural frequency, pull-in voltage, the shift of resonance frequency, and the harmonic response of a ring resonator with eight inside spokes in the form of circular curve beams (rose petals). The numerical modeling and experimental techniques were used for validation. It was found that the natural frequency of silicon [100] is lower than silicon [111] which is due to lower stiffness; however, the pull-in voltage and the shift of frequency of silicon [100] are higher than silicon [111]. The FEM results show an interaction of the local vibration of the ring between spokes and the global vibration of the ring. This local vibration has increased the deviation between the analytical approach and the numerical method for the calculation of the natural frequency to 13.2%. The deviation of FEM and the analytical method for the calculation of pull-in voltage is about 60%, which is due to the assumptions of the analytical method. Shift of resonance frequency increases by increasing bias DC voltage. The harmonic response of the ring reveals that the resonance frequency matches with natural frequency. The prototype is fabricated using the standard SOI based fabrication process and tested to measure the resonance of the ring resonator for different modes. Harmonic results of the experimental and numerical methods show lower deviation since local vibration at both methods is considered. The model approach presented in this paper can be used to provide valuable insights for the optimization of MEMS resonators for various operating conditions.

## Figures and Tables

**Table 1:** Geometrical parameters of the designed ring resonators.

The central angle of electrodes, Δθ	40 deg.
The radius of anchor, $R_{A}$	100 µm
Ring radius (mean), $R_{r}$	615 µm
The radius of the spoke, $R_{S}$	450 µm
The mean radius of the air gap, $R_{g}$	621.5 µm
Width of spoke, $w_{S}$	10 µm
Width of ring, $w_{r}$	10 µm
Thickness of spoke, $h_{S}$	25 µm
Thickness of ring, $h_{r}$	25 µm
Air gap distance, d	3 µm

**Table 2:** Non-dimensional stiffness and effective mass of one circular curve beam.

Non-Dimensional Stiffness		Non-Dimensional Effective Mass	
$\mathrm{Radial}\;\mathrm{Direction},\;\frac{1}{2}k_{sr}^{*}$	$\mathrm{Tan}\mathrm{gential}\;\mathrm{Direction},\;\frac{1}{2}$ $k_{st}^{*}$	$\mathrm{Radial}\;\mathrm{Direction},\;\;\frac{1}{2}m_{sr}^{*}$	$\mathrm{Tan}\mathrm{gential}\;\mathrm{Direction},\;\;\frac{1}{2}m_{st}^{*}$
255.31	6.02	0.18	0.05

**Table 3:** Non-dimensional frequencies of = 2 mode of vibration of the designed ring.

Material	Method	Drive Mode	Sense Mode
Silicon [111]	FEM	4.31	4.31
	Equation (19)	4.88	4.88
Silicon [100]	FEM	4.52	4.63

**Table 4:** Non-dimensional pull-in voltage and pull-in displacement.

Material	Method	$V_{PI}^{*}$	$u_{PI}^{*}$
Silicon [111]	FEM	11.06	0.22
	Equation (22)	14.80	0.33
	Equation (23)	17.75	0.58
Silicon [100]	FEM	10.33	0.13

## Data Availability

Dataset available on request from the authors.

## Appendix A. Electrical Potential Energy

Different groups of electrodes are located around the ring, as driving electrode, sensing electrode, and tuning electrode. The electrical potential energy of each group of electrodes to which the same voltage was applied is as follows:

(A1) $${\left(U_{E}\right)}_{j}=\frac{1}{2}\varepsilon_{0}\varepsilon_{r}\frac{h_{r}R_{g}}{d}{\left[{\left(V_{DC}\right)}_{j}\pm {\left(V_{AC}\right)}_{j}\sin\omega t\right]}^{2}\sum_{i=1}^{{\left(N_{e}\right)}_{j}}\int_{\theta_{i}-\frac{\Delta \theta }{2}}^{\theta_{i}+\frac{\Delta \theta }{2}}\left[1+\frac{u_{r}}{d}+{\left(\frac{u_{r}}{d}\right)}^{2}+{\left(\frac{u_{r}}{d}\right)}^{3}\right]d\theta \;$$

Substituting displacement function, $u_{r}$ from Equation (1) yields to following equation:

$$\begin{array}{cc} {\left(U_{E}\right)}_{j}=\frac{1}{2}\varepsilon_{0}\varepsilon_{r} & \frac{h_{r}R_{g}}{d}{\left[{\left(V_{DC}\right)}_{j}\pm {\left(V_{AC}\right)}_{j}\sin\omega t\right]}^{2}\overset{{\left(N_{e}\right)}_{j}}{\underset{i=1}{\sum }}\{\Delta \theta \\ \; & +\frac{a_{n}}{nd}\left[\sin n\left(\theta_{i}+\frac{\Delta \theta }{2}\right)-\sin n\left(\theta_{i}-\frac{\Delta \theta }{2}\right)\right]\\ \; & -\frac{b_{n}}{nd}\left[\cos n\left(\theta_{i}+\frac{\Delta \theta }{2}\right)-\cos n\left(\theta_{i}-\frac{\Delta \theta }{2}\right)\right]\\ \; & +\frac{{a_{n}}^{2}-{b_{n}}^{2}}{4nd^{2}}\left[\sin2n\left(\theta_{i}+\frac{\Delta \theta }{2}\right)-\sin2n\left(\theta_{i}-\frac{\Delta \theta }{2}\right)\right]+\frac{{a_{n}}^{2}+{b_{n}}^{2}}{2d^{2}}\Delta \theta \\ \; & -\frac{a_{n}b_{n}}{2nd^{2}}\left[\cos2n\left(\theta_{i}+\frac{\Delta \theta }{2}\right)-\cos2n\left(\theta_{i}-\frac{\Delta \theta }{2}\right)\right]\\ \; & +\frac{{a_{n}}^{3}}{nd^{3}}\left[\sin n\left(\theta_{i}+\frac{\Delta \theta }{2}\right)-\sin n\left(\theta_{i}-\frac{\Delta \theta }{2}\right)\right]\\ \; & -\frac{{a_{n}}^{3}}{3nd^{3}}\left[\sin3n\left(\theta_{i}+\frac{\Delta \theta }{2}\right)-\sin^{3}n\left(\theta_{i}-\frac{\Delta \theta }{2}\right)\right]\\ \; & -\frac{{b_{n}}^{3}}{nd^{3}}\left[\cos n\left(\theta_{i}+\frac{\Delta \theta }{2}\right)-\cos n\left(\theta_{i}+\frac{\Delta \theta }{2}\right)\right]\\ \; & +\frac{{b_{n}}^{3}}{3nd^{3}}\left[\cos^{3}n\left(\theta_{i}+\frac{\Delta \theta }{2}\right)-\cos^{3}n\left(\theta_{i}-\frac{\Delta \theta }{2}\right)\right]\\ \; & -\frac{{a_{n}}^{2}b_{n}}{nd^{3}}\left[\cos^{3}n\left(\theta_{i}+\frac{\Delta \theta }{2}\right)-\cos^{3}n\left(\theta_{i}-\frac{\Delta \theta }{2}\right)\right]\\ \; & \left.+\frac{a_{n}{b_{n}}^{2}}{nd^{3}}\left[\sin^{3}n\left(\theta_{i}+\frac{\Delta \theta }{2}\right)-\sin^{3}n\left(\theta_{i}-\frac{\Delta \theta }{2}\right)\right]\right\} \end{array}$$

where *n* is mode number of the vibration. The electrostatic force is defined as derivative of electrical energy in respect to displacement variables:

$${\left(F_{E}\right)}_{j}=\left[\begin{array}{c} -\frac{\partial }{\partial a_{n}}{\left(U_{E}\right)}_{j}\\ -\frac{\partial }{\partial b_{n}}{\left(U_{E}\right)}_{j} \end{array}\right]$$

The electrostatic force in non-dimensional form is expressed as follows:

(A2) $${\left(F_{E}\right)}_{j}=\tau^{2}{{\left(V^{*}\right)}_{j}}^{2}\left\{{\left(k_{E}^{*}\right)}_{j}\left[\begin{array}{c} a_{n}^{*}\\ b_{n}^{*} \end{array}\right]+{\left(d_{1}^{*}\right)}_{j}\left[\begin{array}{c} {\left(a_{n}^{*}\right)}^{2}\\ {\left(b_{n}^{*}\right)}^{2} \end{array}\right]+{\left(d_{2}^{*}\right)}_{j}a_{n}^{*}b_{n}^{*}+{\left(f_{E}^{*}\right)}_{j}\right\}$$

where

(A3) $${\left(f_{E}^{*}\right)}_{j}=\frac{1}{n}d^{*}\left[\begin{array}{c} \sum_{i=1}^{{\left(N_{E}\right)}_{j}}-\left[\sin n\left(\theta_{i}+\frac{\Delta \theta }{2}\right)-\sin n\left(\theta_{i}-\frac{\Delta \theta }{2}\right)\right]\;\\ \sum_{i=1}^{{\left(N_{E}\right)}_{j}}\left[\cos n\left(\theta_{i}+\frac{\Delta \theta }{2}\right)-\cos n\left(\theta_{i}-\frac{\Delta \theta }{2}\right)\right]\; \end{array}\right]$$

$${\left(k_{E}^{*}\right)}_{j}=-{\left(N_{E}\right)}_{j}{\left(\Delta \theta \right)}_{j}\left[\begin{array}{cc} 1 & 0\\ 0 & 1 \end{array}\right]+\frac{1}{2n}$$

(A4) $$\left[\begin{array}{cc} \sum_{i=1}^{{\left(N_{E}\right)}_{j}}-\left[\sin2n\left(\theta_{i}+\frac{\Delta \theta }{2}\right)-\sin2n\left(\theta_{i}-\frac{\Delta \theta }{2}\right)\right]\; & \sum_{i=1}^{{\left(N_{E}\right)}_{j}}\left[\cos2n\left(\theta_{i}+\frac{\Delta \theta }{2}\right)-\cos2n\left(\theta_{i}-\frac{\Delta \theta }{2}\right)\right]\\ \sum_{i=1}^{{\left(N_{E}\right)}_{j}}\left[\cos2n\left(\theta_{i}+\frac{\Delta \theta }{2}\right)-\cos2n\left(\theta_{i}-\frac{\Delta \theta }{2}\right)\right] & \sum_{i=1}^{{\left(N_{E}\right)}_{j}}\left[\sin2n\left(\theta_{i}+\frac{\Delta \theta }{2}\right)-\sin2n\left(\theta_{i}-\frac{\Delta \theta }{2}\right)\right]\; \end{array}\right]$$

$${\left(d_{1}^{*}\right)}_{j}=\frac{3}{nd^{*}}$$

(A5) $$\left[\begin{array}{cc} \overset{{\left(N_{E}\right)}_{j}}{\underset{i=1}{\sum }}-\left[\sin n\left(\theta_{i}+\frac{\Delta \theta }{2}\right)-\sin n\left(\theta_{i}-\frac{\Delta \theta }{2}\right)\right]\; & 0\\ 0 & \overset{{\left(N_{E}\right)}_{j}}{\underset{i=1}{\sum }}\left[\cos n\left(\theta_{i}+\frac{\Delta \theta }{2}\right)-\cos n\left(\theta_{i}-\frac{\Delta \theta }{2}\right)\right]\; \end{array}\right]\;+\frac{1}{nd^{*}}\;\left[\begin{array}{cc} \overset{{\left(N_{E}\right)}_{j}}{\underset{i=1}{\sum }}\left[\sin^{3}n\left(\theta_{i}+\frac{\Delta \theta }{2}\right)-\sin^{3}n\left(\theta_{i}-\frac{\Delta \theta }{2}\right)\right]\; & \overset{{\left(N_{E}\right)}_{j}}{\underset{i=1}{\sum }}-\left[\sin^{3}n\left(\theta_{i}+\frac{\Delta \theta }{2}\right)-\sin^{3}n\left(\theta_{i}-\frac{\Delta \theta }{2}\right)\right]\;\\ \overset{{\left(N_{E}\right)}_{j}}{\underset{i=1}{\sum }}\left[\cos^{3}n\left(\theta_{i}+\frac{\Delta \theta }{2}\right)-\cos^{3}n\left(\theta_{i}-\frac{\Delta \theta }{2}\right)\right]\; & \overset{{\left(N_{E}\right)}_{j}}{\underset{i=1}{\sum }}-\left[\cos^{3}n\left(\theta_{i}+\frac{\Delta \theta }{2}\right)-\cos^{3}n\left(\theta_{i}-\frac{\Delta \theta }{2}\right)\right]\; \end{array}\right]$$

(A6) $${\left(d_{2}^{*}\right)}_{j}=\frac{2}{nd^{*}}\left[\begin{array}{c} \sum_{i=1}^{{\left(N_{E}\right)}_{j}}\left[\cos^{3}n\left(\theta_{i}+\frac{\Delta \theta }{2}\right)-\cos^{3}n\left(\theta_{i}-\frac{\Delta \theta }{2}\right)\right]\;\\ \sum_{i=1}^{{\left(N_{E}\right)}_{j}}-\left[\sin^{3}n\left(\theta_{i}+\frac{\Delta \theta }{2}\right)-\sin^{3}n\left(\theta_{i}-\frac{\Delta \theta }{2}\right)\right]\; \end{array}\right]$$

As seen, the second and third terms in Equation (A2) are nonlinear due to electrostatic forces.

## References

[1] Polunin P.M., Shaw S.W. (2017). Self-induced parametric amplification in ring resonating gyroscopes. Int. J. Non-Linear Mech..

[2] Gill W.A., Howard I., Mazhar I., McKee K. (2022). Development of Starfish-Shaped Two-Ring Microelectromechanical Systems (MEMS) Vibratory Ring Gyroscope with C-Shaped Springs for Higher Sensitivity. Eng. Proc..

[3] Wang D.F., Sagawa T., Lu J., Maeda R. (2012). Analytical study on effect of ring geometry on frequency shift of piezoelectric ring-shaped resonator. Microsyst. Technol..

[4] Liang F., Liang D.-D., Qian Y.-J. (2021). Nonlinear Performance of MEMS Vibratory Ring Gyroscope. Acta Mech. Solida Sin..

[5] Yoon S.W., Lee S., Najafi K. (2011). Vibration sensitivity analysis of MEMS vibratory ring gyroscopes. Sens. Actuators A.

[6] Antonello R., Oboe R., Prandi L., Biganzoli F. (2009). Automatic Mode Matching in MEMS Vibrating Gyroscopes Using Extremum-Seeking Control. IEEE Trans. Ind. Electron..

[7] Amabili M., Paıdoussis M.P. (2003). Review of studies on geometrically nonlinear vibrations and dynamics of circular cylindrical shells and panels, with and without fluid-structure interaction. Appl. Mech. Rev..

[8] Lu T., Tsouvalas A., Mertikine A.V. (2017). The in-plane free vibration of an elastically supported thin ring rotating at high speeds revisited. J. Sound Vib..

[9] Evensen D.A. (1966). Nonlinear Flextural Vibrations of Thin Circular Rings. J. Appl. Mech..

[10] Gallacher B.J., Hedley J., Burdess J.S., Harris A.J., Rickard A., King D.O. (2005). Electrostatic Correction of Structural Imperfections Present in a Microring Gyroscope. J. Micromechanical Syst..

[11] Sieberer S., McWilliam S., Popov A. (2019). Nonlinear electrostatic effects in MEMS ring-based rate sensors under shock excitation shock excitation. Int. J. Mech. Sci..

[12] Gebrel I.F., Wang L., Asokanthan S.F. (2019). Dynamic Analysis and Design of a Novel Ring-Based Vibratory Energy Harvester. Vibration.

[13] Leissa A.W. Non-linear Analysis of Plates and Shell Vibration. Proceedings of the Second International Conference on Recent Advances in Structure Dynamics.

[14] Moussaoui F., Benamar R. (2002). Non-linear Vibrations of Shell-type Structures: A Review with Bibliography. J. Sound Vib..

[15] Polunin P., Yang Y., Atalaya J., Ng E., Strachan S., Shoshani O., Dykman M., Shaw S., Kenny T. Characterizing MEMS Nonlinearities Directly: The Ring-down Measurements. Proceedings of the 2015 Transducers—2015 18th International Conference on Solid-State Sensors, Actuators and Microsystems (TRANSDUCERS).

[16] Hu Z.X., Gallacher B.J., Burdess J.S., Bowles S.R., Grigg H.D. (2014). A systematic approach for precision electrostatic mode tuning of a MEMS gyroscope. J. Micromechanics Microengineering.

[17] Yu T., Kou J., Hu Y.-C. (2018). Vibration of a Rotating Micro-Ring under Electrical Field Based on Inextensible Approximation. Sensors.

[18] Asokanthan S.F., Cho J. (2006). Dynamic stability of ring-based angular rate sensors. J. Sound Vib..

[19] Timoshenko S. (1937). Vibration Problems in Engineering.

[20] Ranji A.R., Guo J., Alirezaee S., Ahamed M.J. (2023). Modelling and dynamic analysis of a MEMS ring resonator supported by circular curved shaped inner beams. Phys. Scr..

[21] Acar C., Shkel A. (2009). MEMS Vibratory Gyroscopes, Structural Approaches to Improve Robustness.

[22] Becker R.A. (1954). Introduction to Theoretical Mechanics.

[23] Ranji A.R., Li A., Alirezaee S., Ahamed M.J. (2023). Analytical modeling of an inclined folded-beam spring used in micromechanical resonator devices. Eng. Res. Express.

[24] ANSYS (2022). Advanced Analysis Guide.

[25] Zhang S., Zhang W.-M., Peng Z.-K., Meng G. (2015). Dynamic Characteristics of Electrostatically Actuated Shape Optimized Variable Geometry Microbeam. Shock. Vib..

[26] Fischer M., Giousouf M., Schaepperle J., Eichner D., Weinmann M., Munch W.V., Assmus F. (1998). Electrostatically deflectable polysilicon micromirrors—Dynamic behaviour and comparison with the results from FEM modelling with ANSYS. Sens. Actuators A.

[27] Hu Y.C., Chang C.M., Huang S.C. (2004). Some design considerations on the electrostatically actuated microstructures. Sens. Actuators A.

[28] Bao M. (2005). Electrostatic Actuation. Analysis and Design Principles of MEMS Devises.

[29] Mohamad N., Iovnitti P., Vinay T. Effective Diaphragm Area of Spring-Supported Capacitive MEMS microphone Designs. Proceedings of the SPIE Smart Materials, Nano- and Micro-Smart Systems.

[30] Eley R., Fox C.H., McWilliam S. (1999). Anisotropy Effects on the Vibration of Circular Rings Made from Crystalline Silicon. J. Sound Vib..

[31] Shirin G., Ahn C., Ng E., Wang S., Kenny T. Silicon Crystal Effects in Modeling of MEMS Silicon Resonators. Proceedings of the NSTI-Nanotech 2012 NSTI Nanotechnology Conference and Expo.

[32] Jia J., Ding X., Qin Z., Ruan Z., Li W., Liu X., Li H. (2021). Overview and analysis of MEMS Coriolis vibratory ring gyroscope. Measurment.

[33] Qin Z., Gao Y., Jia J., Ding X., Huang L., Li H. (2019). The Effect of the Anisotropy of Single Crystal Silicon on the Frequency Split of Vibrating Ring Gyroscopes. Micromachines.

